# Supplementary figures and images for: BARcode DEmixing through Non-negative Spatial Regression (BarDensr)
Source: PLoS Comput Biol. 2021 Mar 8;17(3):e1008256. doi: 10.1371/journal.pcbi.1008256 (PMC7971881; doi:10.1371/journal.pcbi.1008256)

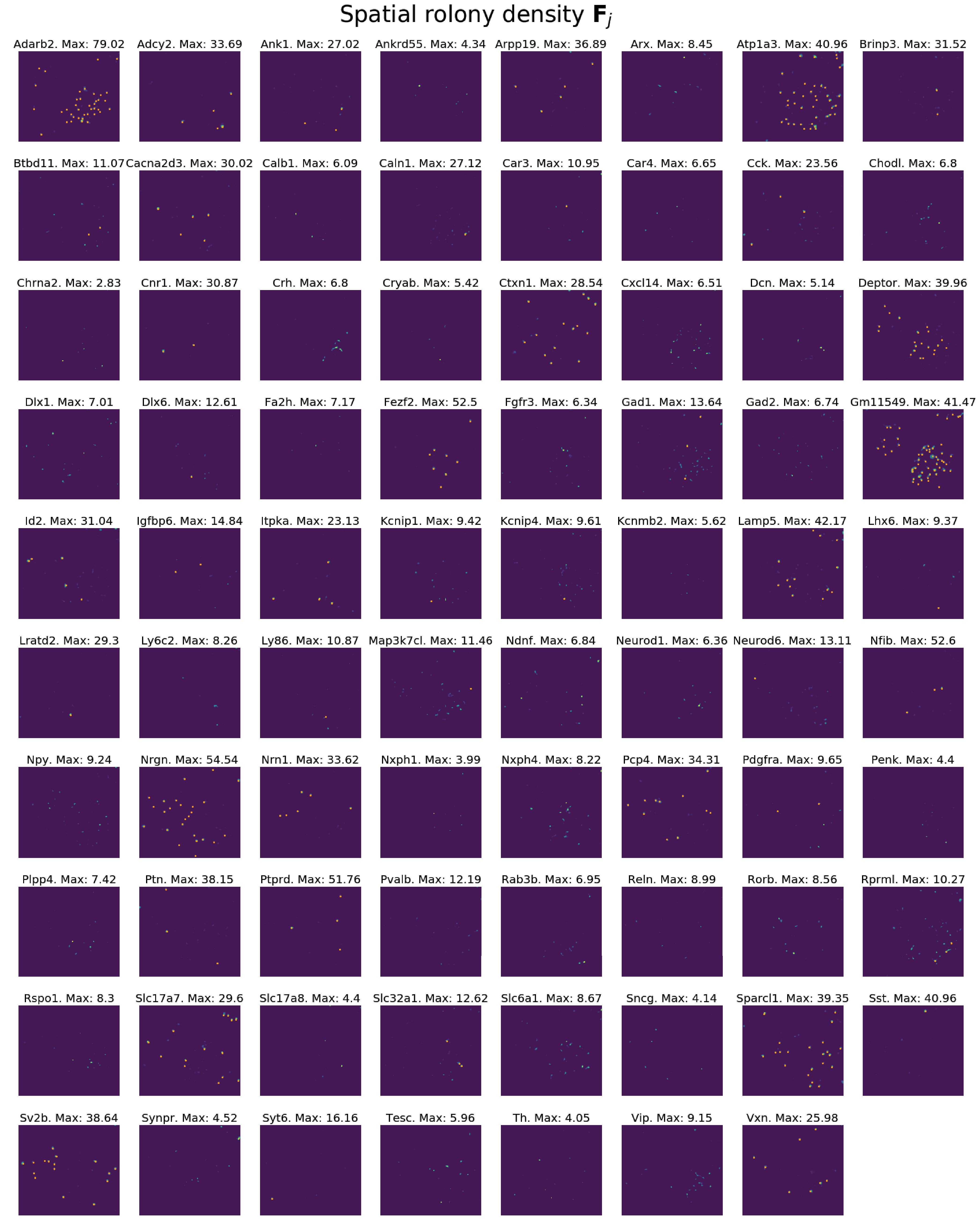

Supplement: S1 Fig — These images are the supplement to Fig 2A in the main text. The rolony densities represent a demixed view of the data. Each plot corresponds to a single barcode, and indicates the rolony density at different spatial locations. Above we show these rolony densities for one region in the experimental data. The title for the plots above indicates the gene associated with the barcode as well as the maximum intensity of the plot. The orange dots represent rolonies detected by a hand-curated approach. (TIF) [file pcbi.1008256.s002.tif]

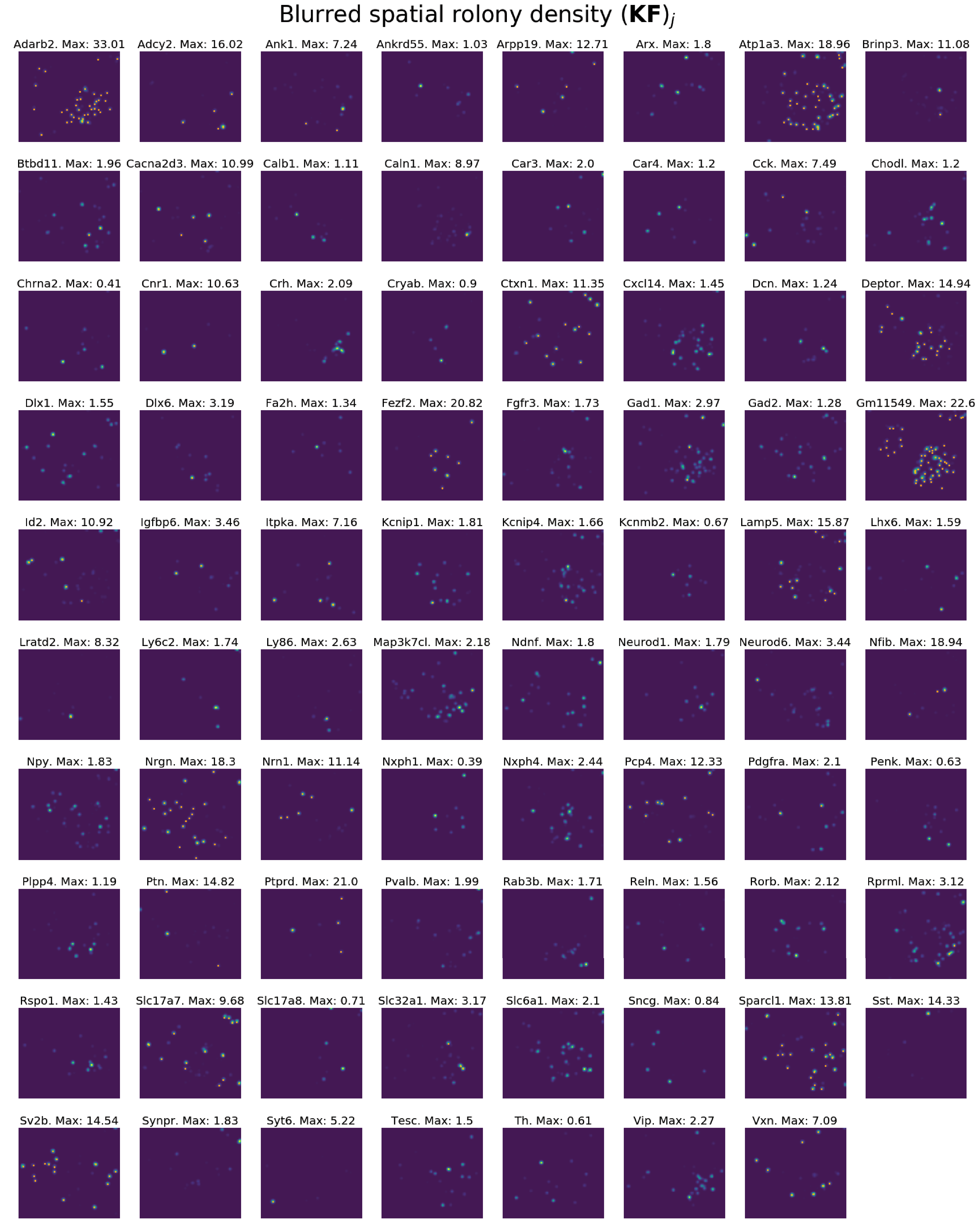

Supplement: S2 Fig — As of S1 Fig, these images are the supplement to Fig 2A in the main text, except we display (KF)j instead of (F)j for each barcode j. Recall that the point-spread function K has the effect of smearing signal over a spatially localized area. It represents physical processes which blur the signal of interest. Under the BarDensr model, the signal intensities observed at each voxel m from a given barcode will arise directly from linear combinations of (KF)m,j over different barcodes j. (TIF) [file pcbi.1008256.s003.tif]

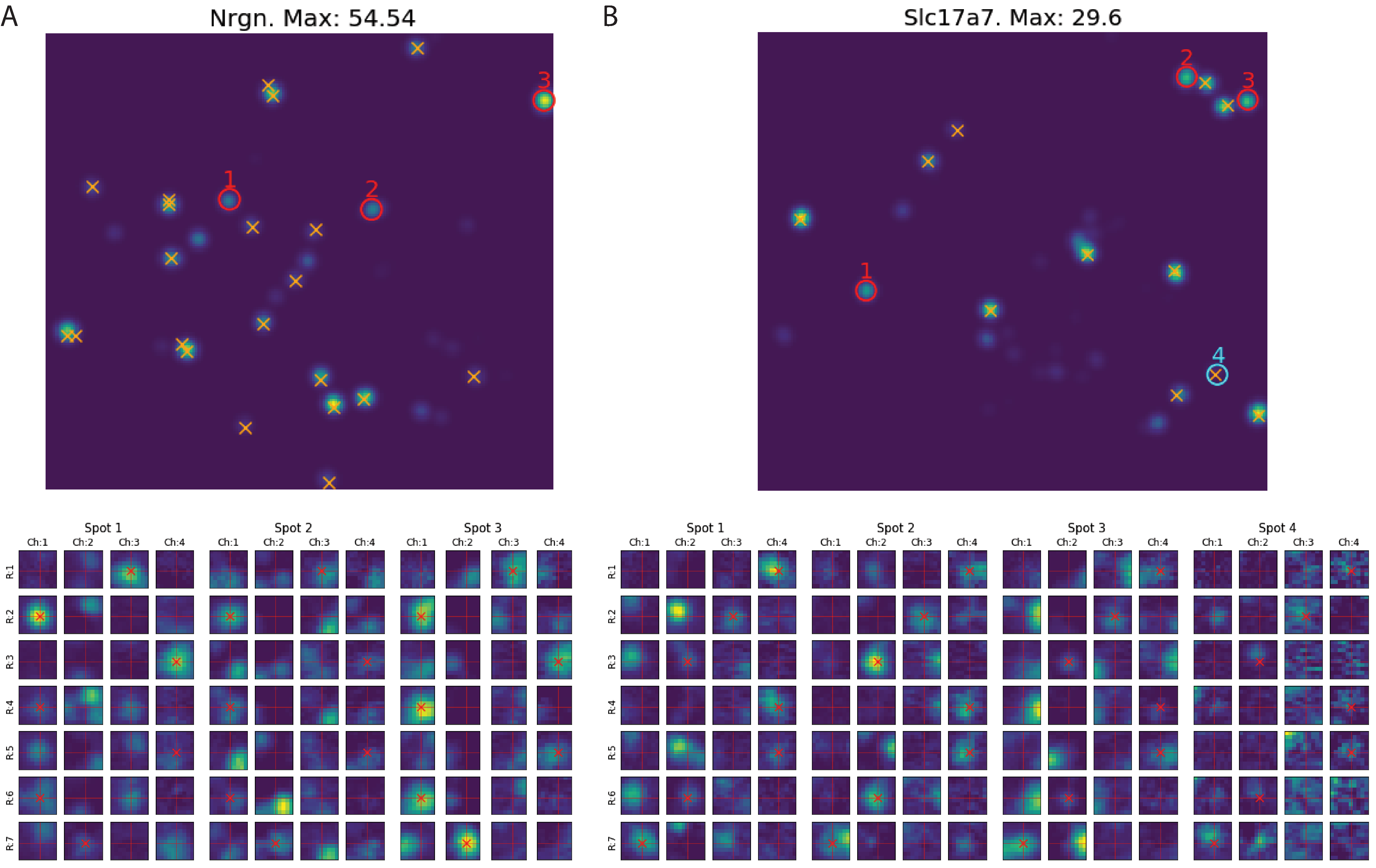

Supplement: S3 Fig — (A) Nrgn. The top plot shows the same rolony density of Nrgn as in Fig 2A. The orange crosses indicate the spots detected in the hand-curated results. The three spots highlighted with red are further zoomed in the bottom. These spots were detected to have large signal intensities by BarDensr, but were not detected in the hand-curated results. The correct barcode frames for Nrgn are indicated with red crosses in the bottom plots, suggesting that each of these spots appear to be well-modeled as Nrgn spots. (B) Slc17a7. The top plot shows the same rolony density of Slc17a7 as in Fig 2A. The orange crosses indicate spots detected by hand-curated method. The four spots highlighted with red or cyan are further zoomed in the bottom. The first three spots (Spot 1—3, shown in red) were found by BarDensr but were not detected by hand-curated results. The fourth spot (Spot 4, shown in cyan) is the spot that is detected by hand-curated results, but no signal detected in BarDensr. The correct barcode frames for Slc17a7 are indicated with red crosses in the bottom plots. (TIF) [file pcbi.1008256.s004.tif]

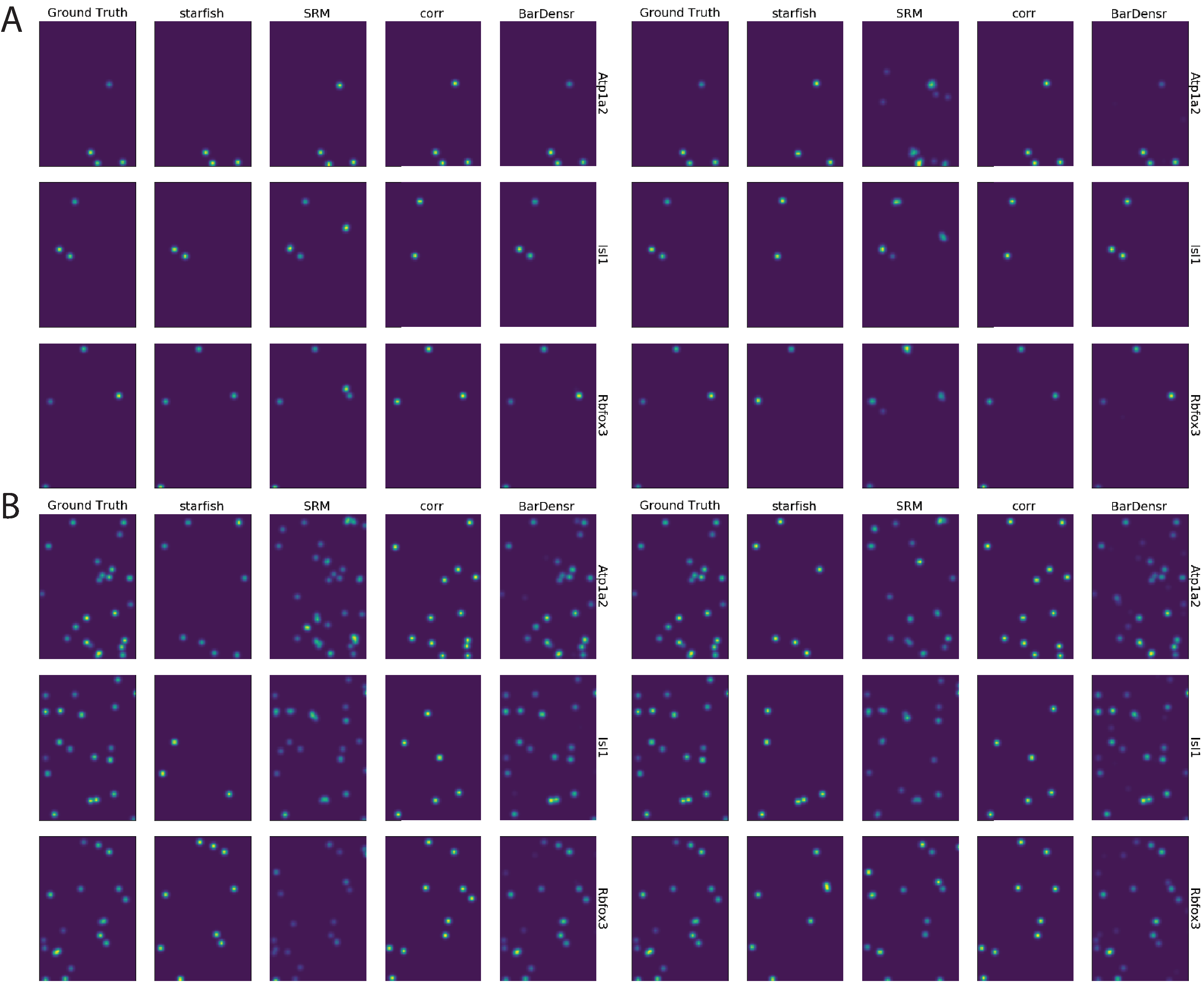

Supplement: S4 Fig — (A) Benchmarking results on the regular simulation. Comparing starfish, SRM, ‘corr’, and BarDensr results, to the ground truth. Showing the top three barcodes with highest density (the gene density was generated randomly, see S1 Appendix, Section G). The left panel is with no dropout, and it corresponds to the top left plot in Fig 3A in the main text. Without dropout, BarDensr accurately detects the barcodes in the original data. The right panel is similar to the left, but with dropout for 50% of the simulated spots. This corresponds to the top right plot in Fig 3A. (B) Benchmarking results using five times denser simulation. This is similar except that the spots density is five times denser than (A). The left and right panels are without and with dropout, as explained earlier, and they correspond to the bottom left and bottom right in Fig 3A, respectively. With dropout for 50% of the densely simulated spots, some missing spots (FN) can be observed from these methods (e.g., see the first row Atp1a2). False discovery (FP) can also be seen in this plot for SRM (e.g., see the third row Rbfox3). (TIF) [file pcbi.1008256.s005.tif]

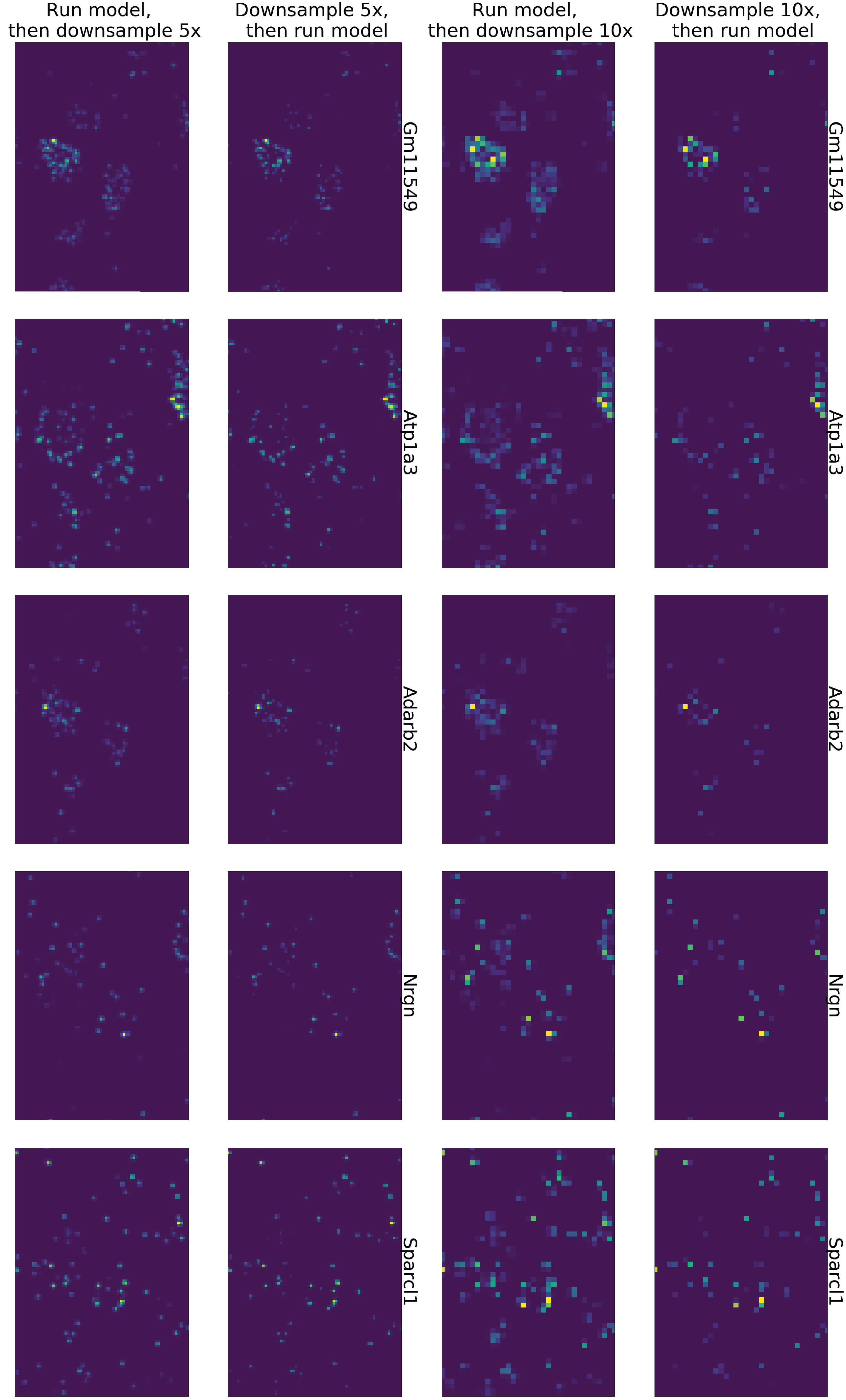

Supplement: S5 Fig — To test BarDensr’s performance on low-resolution data, we first run BarDensr on the original data, obtain rolony densities, and then finally downsample the rolony densities (‘run-then-downsample’). Next, we run BarDensr on downsampled data and look at the learned rolony densities (‘downsample-then-run’). For highly-expressed genes, these two results are nearly indistinguishable. (TIF) [file pcbi.1008256.s006.tif]

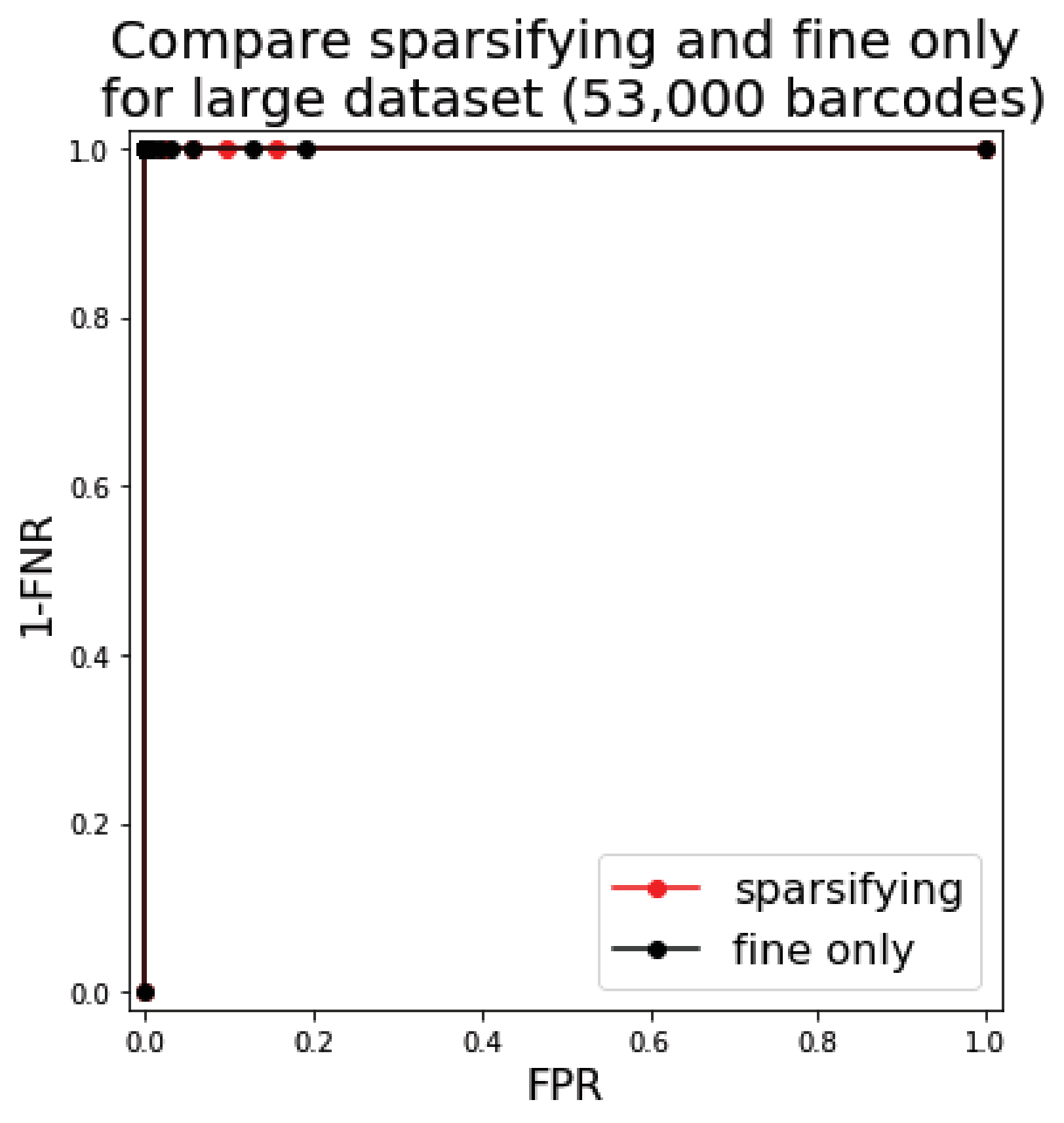

Supplement: S6 Fig — To test if we can scale up BarDensr, we computed an ROC curve for the method using a simulated dataset with 53,000 barcodes and 17 sequencing rounds. After running the model on a 5× downsampled 50 × 80 voxels simulated image, barcodes that are set to zero at the coarse scale were removed and the model was run at the original scale, with the parameters learned from the downsampled image as the initial conditions. See also S1 Appendix (Section K). (TIF) [file pcbi.1008256.s007.tif]

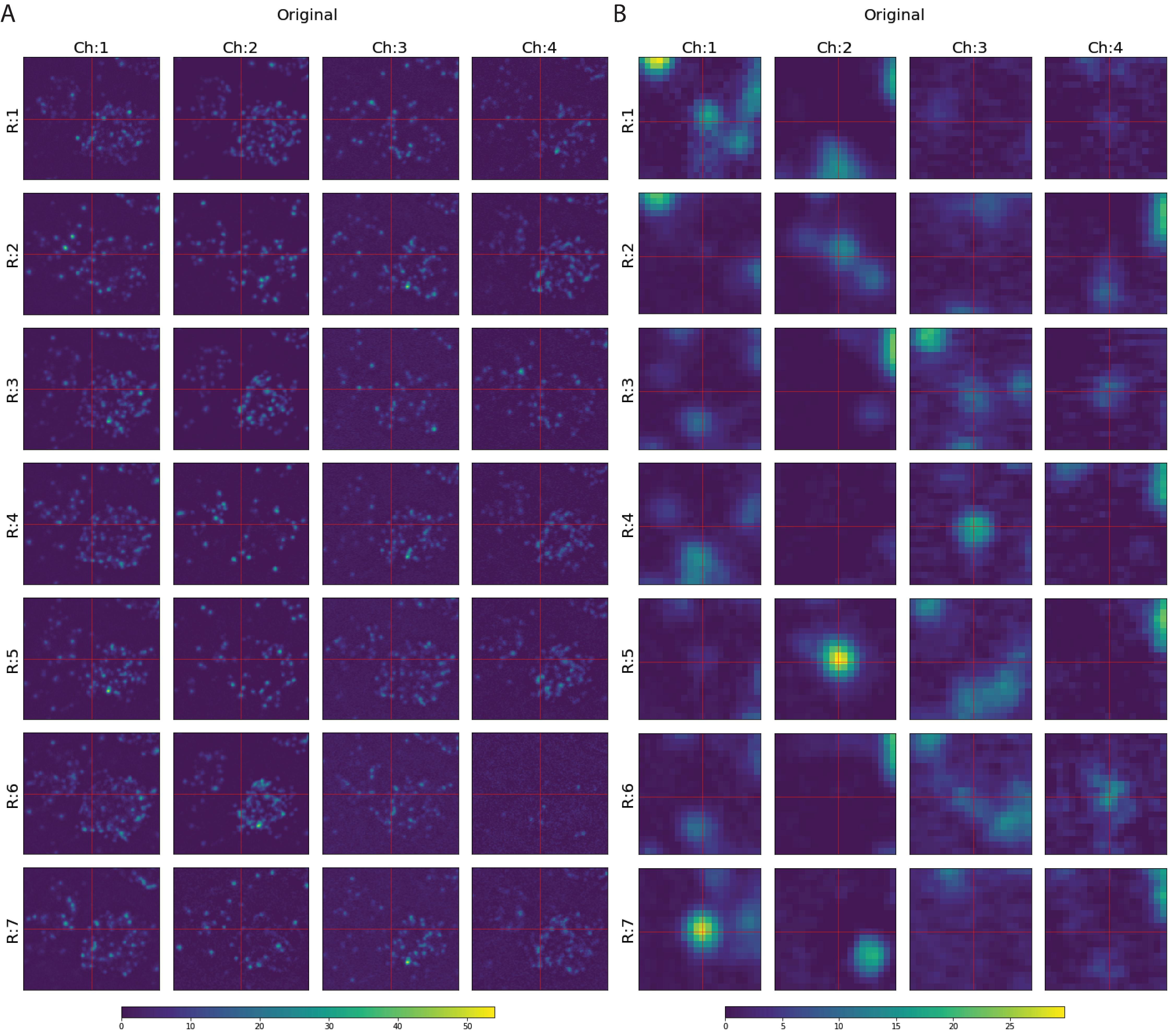

Supplement: S7 Fig — In order to create clearer visualizations, we noise-normalized the data as described in S1 Appendix (Section A), so that images from all rounds and channels are on the same scale. (A) shows the zoomed-out images in the selected region. (B) shows the zoomed-in images for one of the target spots (a 20 × 20 region). Also see the video visualization at https://tinyurl.com/y7zzyrd4. (TIF) [file pcbi.1008256.s008.tif]

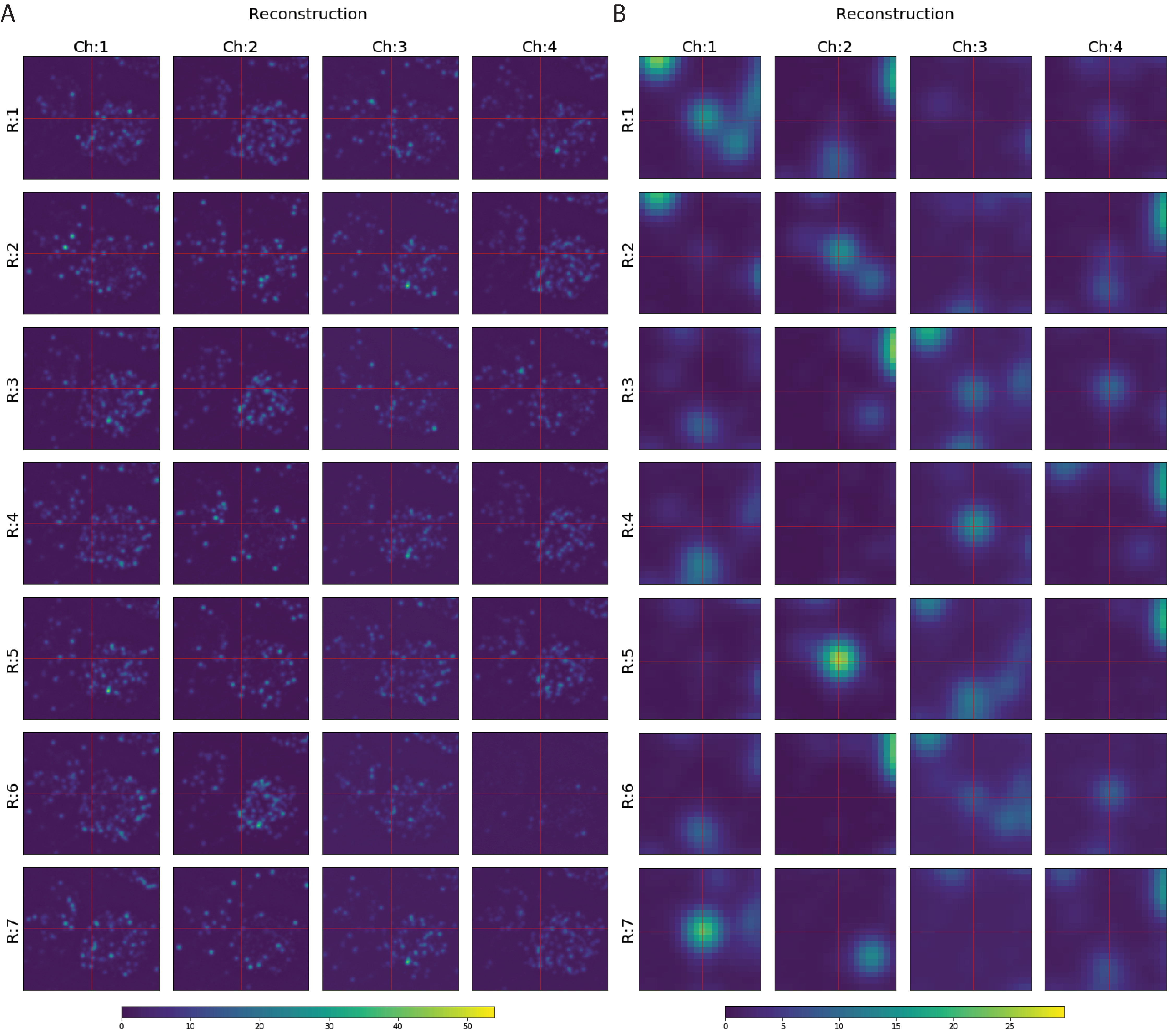

Supplement: S8 Fig — Under the BarDensr model, the fluorescence signal observed at each voxel in S7 Fig. should be approximately given by the equations from the Methods Section. We here plot the results of those equations, visualized using the same colormap-intensity scale as used in S7 Fig. At least by eye, we see excellent agreement between the data and the model’s predictions. (A) and (B) are zoomed-out and zoomed-in images as described in S7 Fig. Also see the video visualization at https://tinyurl.com/y7zzyrd4. (TIF) [file pcbi.1008256.s009.tif]

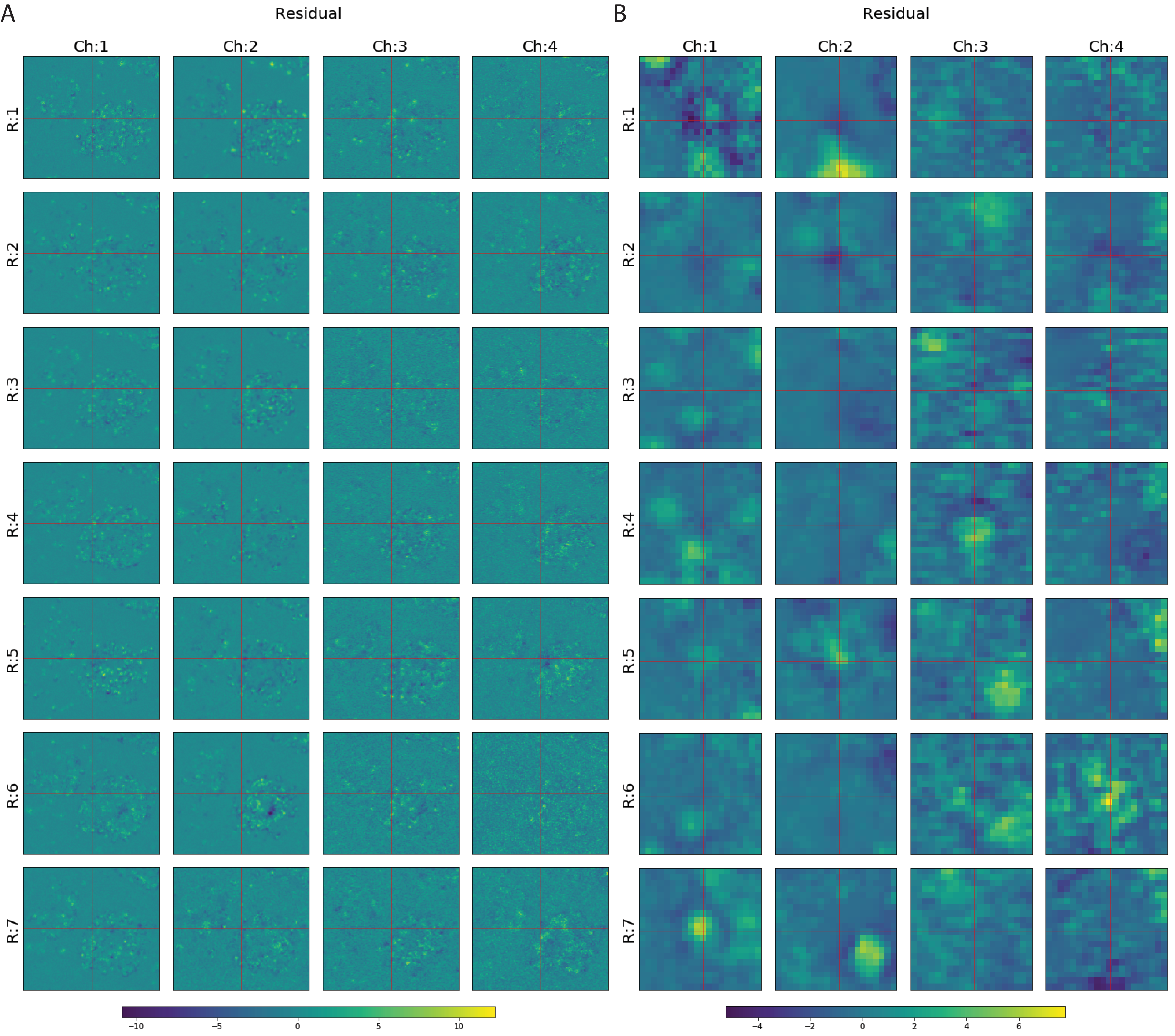

Supplement: S9 Fig — As mentioned in S8 Fig, the BarDensr model makes predictions about what the observed data should look like. There is broad agreement, but there is some disagreement. Here we highlight the the residual between the predictions and the data. Note the difference in scale compared to the previous two figures. (A) and (B) are zoomed-out and zoomed-in images as described earlier. Also see the video visualization at https://tinyurl.com/y7zzyrd4. (TIF) [file pcbi.1008256.s010.tif]

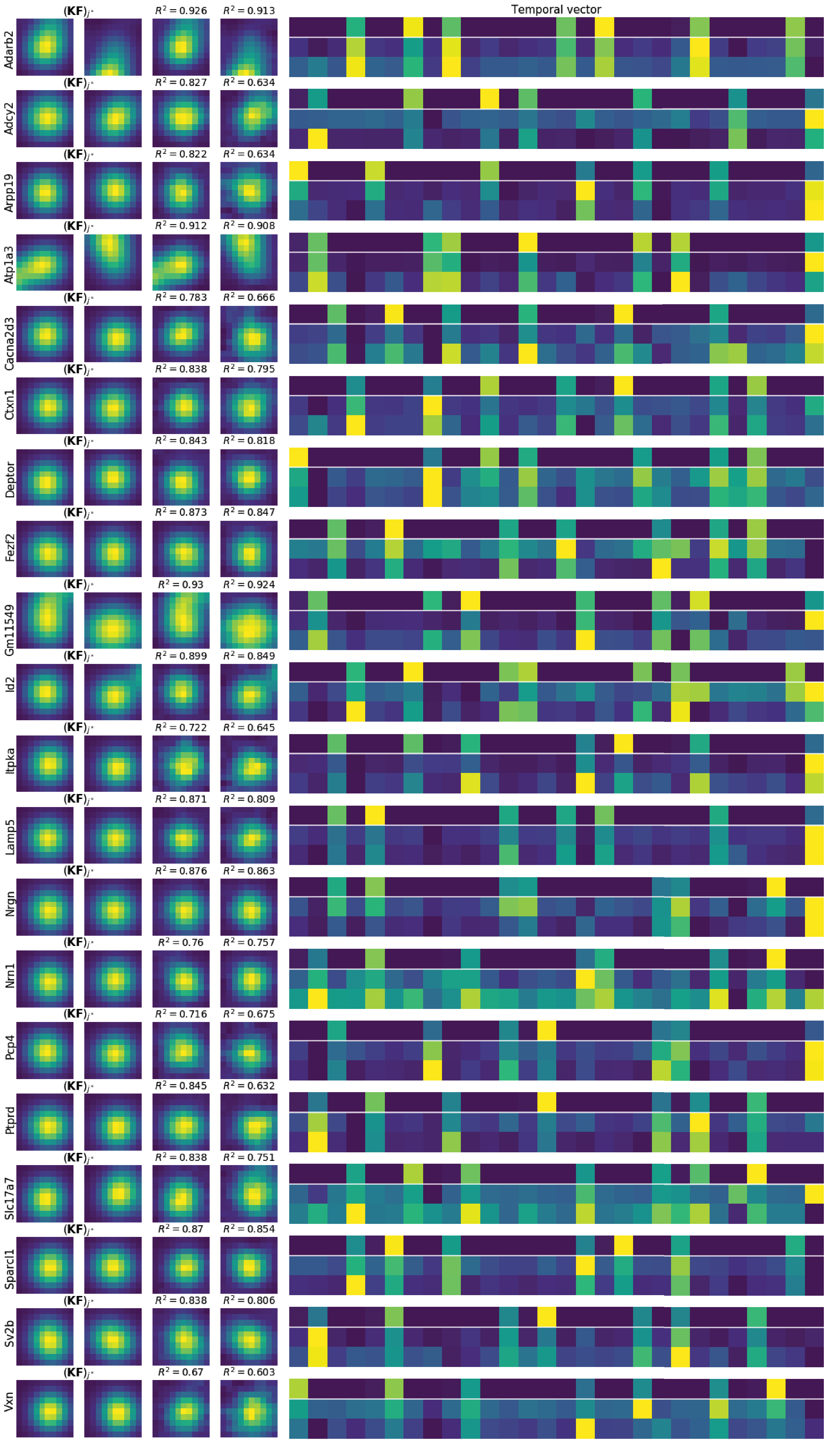

Supplement: S10 Fig — The figure supplements Fig 7 in the main text, and is structured in the same way, except that this plot shows more examples (with more barcodes and spots). Each row shows two spots for a given barcode. The first two columns show (KF)j* cropped around the two spots; the third and forth columns show the top spatial singular vectors for the same crops. The final wide column shows the top temporal singular vectors for these spots, with the first row (above the thin white line) showing the scaled Gj* learned from the model, and the following two rows showing the corresponding top temporal singular vectors for these spots. The two spots are ordered by R2, which is computed as in Fig 7. (TIF) [file pcbi.1008256.s011.tif]

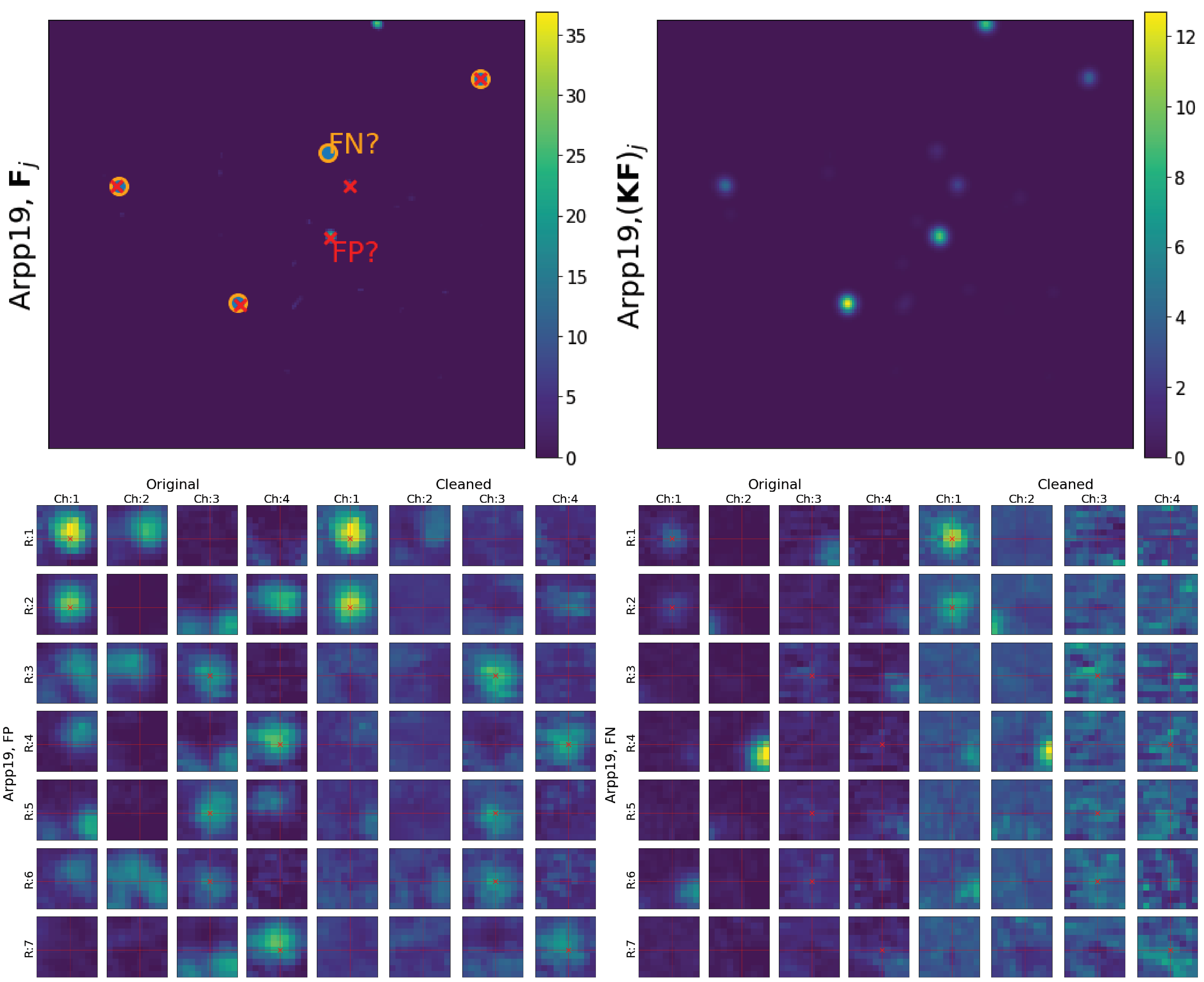

Supplement: S11 Fig — On the top two plots, we show the rolony density Fj (left) and the blurred rolony density (KF)j (right) for gene Arpp19, derived from the experimental data. These rolony densities indicate the presence of Arpp19-rolonies. However, they might be incorrect, indicating that these detected rolonies might not be present in the real data. In this figure we investigate this question qualitatively. First, we compare with the rolony positions detected by a hand-curated method (as represented by orange circles on the left top plot) with the rolonies suggested by the rolony densities (as indicated by red crosses on the left top plot). We see a broad agreement. Where there is a point of disagreement, we can visualize the signal intensities in all the voxels near that point. The two plots on the bottom-left show the original data from a spot that was detected by BarDensr, but not detected in the hand-curated results (as indicated as False Positive (FP) in the top left plot); the left columns show the original image and the right columns show the ‘cleaned’ image (similar to Fig 7, see Eq 2 for details). The red cross in each round indicates the channels that are activated by this barcode. These crosses line up well with the observed signal, suggesting BarDensr has correctly identified a new rolony. It appears that the hand-curated method failed to detect this rolony because of the presence of nearby rolonies, leading to a mixed signal; BarDensr is specifically designed to handle these kinds of confusing situations. The two plots on the bottom-right show a spot which is detected in the hand-curated result but not detected by BarDensr (as indicated as False Negative (FN) in the top left plot). We show both the original data and the cleaned data, as in the bottom left plots. In this case, the data do not appear to support the presence of a rolony, suggesting BarDensr correctly rejected this region as a rolony and the the hand-curated approach labeled it incorrectly. We conjecture th [file pcbi.1008256.s012.tif]
