# Supplementary material for: BARcode DEmixing through Non-negative Spatial Regression (BarDensr)
Source: PLoS Comput Biol. 2021 Mar 8;17(3):e1008256. doi: 10.1371/journal.pcbi.1008256 (PMC7971881; doi:10.1371/journal.pcbi.1008256)
Supplement: Attachment — Submitted filename: response_to_reviewers.pdf [file pcbi.1008256.s013.pdf]

January 7, 2021

Dear Editor-in-Chief,

We thank the reviewers for their constructive feedback on our manuscript, which led to two major changes. First, we created a more rigorous benchmark; unfortunately, no public benchmark yet exists for this kind of data, to our knowledge, so we painstakingly annotated a region of data by hand to get a more quantitative assessment of performance. Second, we demonstrated the performance of the method on a larger region of mouse motor cortex, allowing readers to see how BarDensr is able to identify spatial patterns in the data. In addition to these main changes, we have clarified that the barcode list used to generated the simulation data in Figure 4 and Figure S10 - S13 will be publically available (line 666), as required by the PLOS data access policy. There were also several other notable points, which we address below. All changes in our new manuscript (attached) are highlighted with purple.

Again, thank you for your consideration and we look forward to hearing from you.

Sincerely,
Shuonan Chen

Reviewer 1

"In figure 4 and specially Figure 5, when assessing the performance of BarDensr compared to other methods, in all the situations BarDensr performs well. One would maybe like to see, especially in figure 5, how far can one go and when the method starts to fail, by adding more extreme cases with even more dense spots in the simulated data. "
Good point; we have increased the maximum number of spots in the hybrid simulations from 200 to 2000. At this extreme level, the SRM method fails completely and BarDensr begins to fail (especially in the case with dropout).

"When discussing how computationally expensive this method is, it would be good to do that in comparison with the other methods used to benchmark the method."
Agreed. We have included this time comparison into the results section when we described the newly added Figure 7 (where we compare BarDensr with other methods using the benchmark data at the original scale). The other methods are indeed faster than BarDensr (though not prohibitively so) and we consider this to be the main caveat of using BarDensr, although we provided several approaches to speedup the computation.

"One of the main problems when analyzing image-based spatially resolved RNA sequencing methods are stitching problems and misalignments between cycles. It would be good to include a paragraph about this."
These problems are indeed important, but they are not the problems that BarDensr is designed to resolve. Best practices for these problems are often quite dependent on the exact detail of the experimental design. In future work we hope to potentially resolve these problems. We have added some discussion on this in Section 4 (*Discussion*, line 464).

"Here and there, I got lost when navigating between main figures and supplementary ones. I would prefer is more of the illustrations found in the supplementary figures could be added to the main figures."
We have improved the hyperlinking in the pdf so that it should be easier to navigate. Unfortunately, we could not find any way to include more supplementary figures without utterly overwhelming the main text.

"As a molecular biologist, I would have liked to see the method applied to larger piece of a biological specimen, like a region of the mouse brain displaying a high diversity of cell types, just to see what improvements can be achieved in terms of number of transcripts detected per cell, and whether the distribution of cell-type specific markers become tighter (and less dispersed), the letter being an indicator of improved specificity in barcode identification."
We have applied BarDensr to a large section of motor cortex and the result was added into Figure 10. In this new figure, we are able to see the striation of the cortex. The data was obtained from a recent study by Sun et al., 2020, as explained in the data description section (Section 2, line 66) in the updated paper.

Regarding transcripts detected per cell – on this dataset, spot density is modest, and we found that BarDensr did not significantly outperform other state-of-the-art methods. However, if we increase the spot density per pixel, by blurring and downsampling, then BarDensr does improve on the state of the art, as expected. We have additionally included a new hand-crafted benchmark using real data, giving a clearer understanding of how different methods perform in different situations.

Regarding tighter markers – based on the marker genes identified by BarDensr, we estimated the cell types for each cell (as shown in Figure 10). For each type, we then estimated the entropy of the distributions of the locations of the corresponding spots. BarDensr revealed that some cell types have even tighter spatial distributions than demonstrated by the original study's methodology. This suggests BarDensr obtains superior barcode identification. However, the difference is not very great, and we decided not to include it in the paper.

Reviewer 2

"1. The use of "spatial regression" in the title should be removed as it may be confusing for readers who may think this approach uses spatial models whereas the current paper uses non-negative least squares."
Thank you for the suggestion. However, BarDensr is indeed a spatial model (the point-spread operator $\mathbf{K}$ creates a spatial dependence among all the variables), so we decided to keep spatial regression in the title.

"2. The authors used a partial subtraction approach to assess barcode-specific contribution. However, the overall reconstruction error should be reported using the flattened vector containing the observed pixel values."
We have computed the overall reconstruction error in units of percentage unexplained (we found results of around 5-10% being typical), and added this quantification in the result section "*The BarDensr model correctly predicts the observed signal intensities*" in the updated manuscript line 411.

"3. Since the current framework uses some tensorflow functions under the hood, I am wondering how this approach would compare to ANNs and CNNs? Have the authors compared the present methodologies to neural network approaches based solely on reconstruction error as well as computation times?"
We were unable to find any useable tools attempting to solve these problems with neural networks. It is not clear what the best approach might be. It would be interesting for a future investigation, and we have added a short discussion in Section 4 (*Discussion*, line 468) – we would be curious to see if they can perform better than our regression model.

"4. The google collab notebook fails when generating the rez object (rez=bardensir.singlefov.process()) - "using a 'tf.Tensor' as a Python 'bool' is not allowed:""
This was caused by a code conflict related to the 2.3.0 update of Tensorflow. We believe it is fixed now. Thank you for pointing out.

"5. Are the 28 Figures supposed to be part of the main manuscript? Is there a reason for not labelling some as supplemental figures (e.g. Supplementary Figure 1 etc)?"
We have fixed the labels and now there are 12 main figures (2 of them are newly added for the re-submission) and 18 supplementary figures. The supplementary figures are labeled as Figure S1, Figure S2, ... instead.

Reviewer 3

"1. While I am not in favor of over-simplifying methods descriptions, I feel it would benefit your user

community to briefly outline the steps utilized in a typical BarDensr workflow very specifically from the perspective of the user. This should include when to employ additional features in the workflow, such as interactive estimation of omega or estimation of good fit."

Thank you for the suggestion. At this point, we do not consider BarDensr as being a workflow *in itself*. It does not include many crucial steps which are necessary to create a complete product (e.g. image registration, denoising, cell-identification). Unlike a full image processing pipeline such as "*starfish*," we consider BarDensr a tool that can be used as part of a pipeline. In other words, the target audience for the BarDensr package is data-scientists who intend to create workflows for experimentalists, rather than experimentalists themselves. We have added a description in Section 1 (*Introduction*, line 48) to clarify the scope of our method.

"2. Since data preprocessing will probably be an important consideration in a typical analysis: Are there particular data preprocessing approaches that are optimal for satisfactory BarDensr analysis? How would a user estimate performance, perhaps with the SVD approach shown in Fig 10? Is there a suggested way to iterate to find the best set of parameters for data preprocessing?"

We decided to leave the preprocessing steps for the users. Input images are assumed to be filtered, well-registered and corrected for potential artifacts whenever is possible, although BarDensr can account for some of these artifacts. This is because the necessary preprocessing steps are likely dependent on the experimental platform and conditions (information that is hard to be passed on to a computational tool!). That being said, we did find that noise-normalization is crucial in order to get a reasonable result, and this step has been described in Appendix A. We have also added a new paragraph in the same section describing the normalization on the spot intensity across frames, which we also have found to be helpful (line 578).

Regarding how to choose the best parameters, we have found it useful to iteratively examine results on small patches of data and then scale up to full datasets. The "cleaned images" we describe in the paper do give some quantitative assessment that helps this process – these cleaned images should be approximately rank-1 when considered as matrices, and large deviations from rank-1 (as computed by SVD) indicate something might be going wrong. Following your suggestions, we now describe this idea in more detail in the last section in Section 2 (*Code availability*, line 253).
