# Supplementary material for: BARcode DEmixing through Non-negative Spatial Regression (BarDensr)
Source: PLoS Comput Biol. 2021 Mar 8;17(3):e1008256. doi: 10.1371/journal.pcbi.1008256 (PMC7971881; doi:10.1371/journal.pcbi.1008256)
Supplement: Attachment — Submitted filename: response_to_reviewers_2.pdf [file pcbi.1008256.s014.pdf]

February 3, 2021

Dear Editor-in-Chief,

We thank the reviewers for their feedback on our manuscript. For this re-submission we mainly reflected the suggestion on figure arrangement from Reviewer one, as described in detail below. All text changes in our new manuscript (attached) are highlighted with brown, in addition to the purple from the last submission.

Again, thank you for your consideration and we look forward to hearing from you.

Sincerely,
Shuonan Chen

Reviewer 1

“The authors have adequately responded to the comments and questions. One of the points of criticism is the arrangement of the figures. There are 12 main figures, some with very little content and then 18 supplemental figures, whereof many are really repetitive in nature. To increase the readability, I would suggest that some figures are merged (eg. 1+2+3; 4+5; and 7+8+9). Many of the repetitive elements of the supplementary figures could be reduced in order also to merge some of them. ”

Thank you for the suggestion. Following your suggestion, we have merged the figures to improve the readability. Specifically, we have merged the original Figure 2 and 3 (into the new Figure 2), original Figure 4 and 5 (into the new Figure 3), original Figure 6 and 7 (into the new Figure 4), original Figure 8 and 9 (into the new Figure 5), and original Figure 11 and 12 (into the new Figure 7). This reduced the number of main figures to 7. Similarly, for the supplementary figures, we have merged Figure S3-S8 into two figures. Figures S10-S13 are also merged into one figure. This has reduced the total number of supplementary figures to 11. We were able to reduce most of the repetitive contents in the figures by merging them. However, Figure 1 was too large to be merged into Figure 2 and 3. For the merged figures, we also made the figure legends more succinct, which are all highlighted in brown, and rearranged the order when necessary.

Reviewer 3

“Thank you for the changes. In the response, you wrote that your method is not a pipeline. I meant as your ”user base” being actual bioinformaticians who may want to tweak parameters. But I understand what you’re saying about dropping it into a pipeline.”

Thank you for the clarification. We agree that it would be helpful for the analyst to be able to tweak the parameters. The information on the parameters that can be tweaked could be found in the NeuroCAAS page as well as our Appendix C.
